# Supplementary material for: Long-Term Evaluation of Retinal Morphology and Function in Rosa26-Cas9 Knock-In Mice
Source: Int J Mol Sci. 2023 Mar 8;24(6):5186. doi: 10.3390/ijms24065186 (PMC10049241; doi:10.3390/ijms24065186)
Supplement: Supplementary file 1 [file ijms-24-05186-s001.zip › ijms-2182490-supplementary.pdf]

Supplementary Figure S1

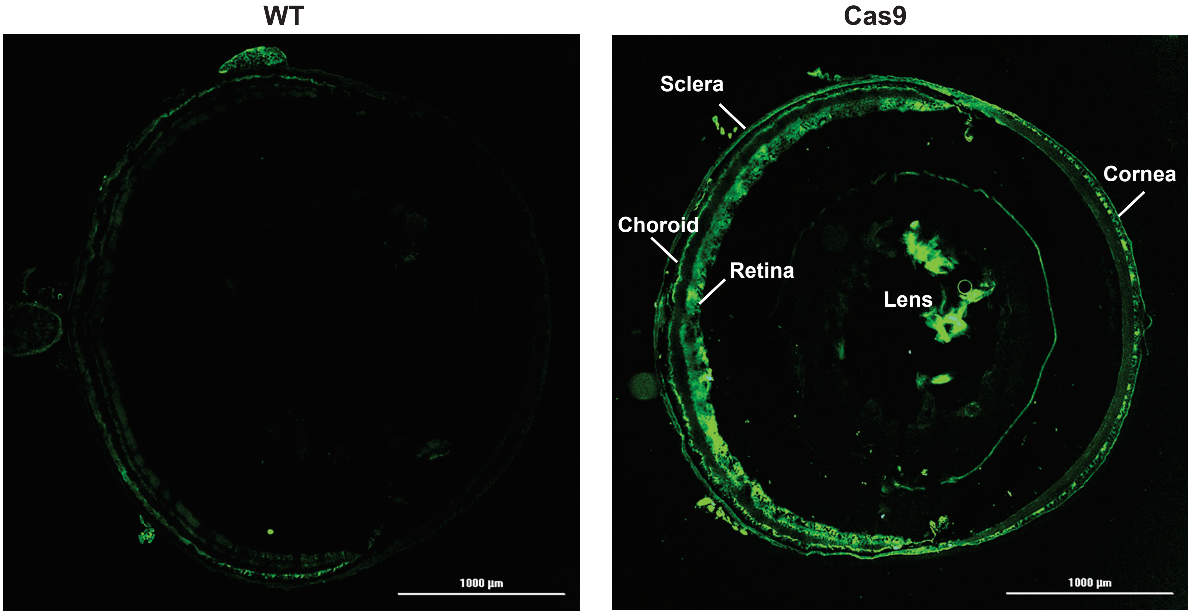

Figure S1: Whole mount fluorescence of WT and Cas9 mouse eye

Supplementary Figure S2

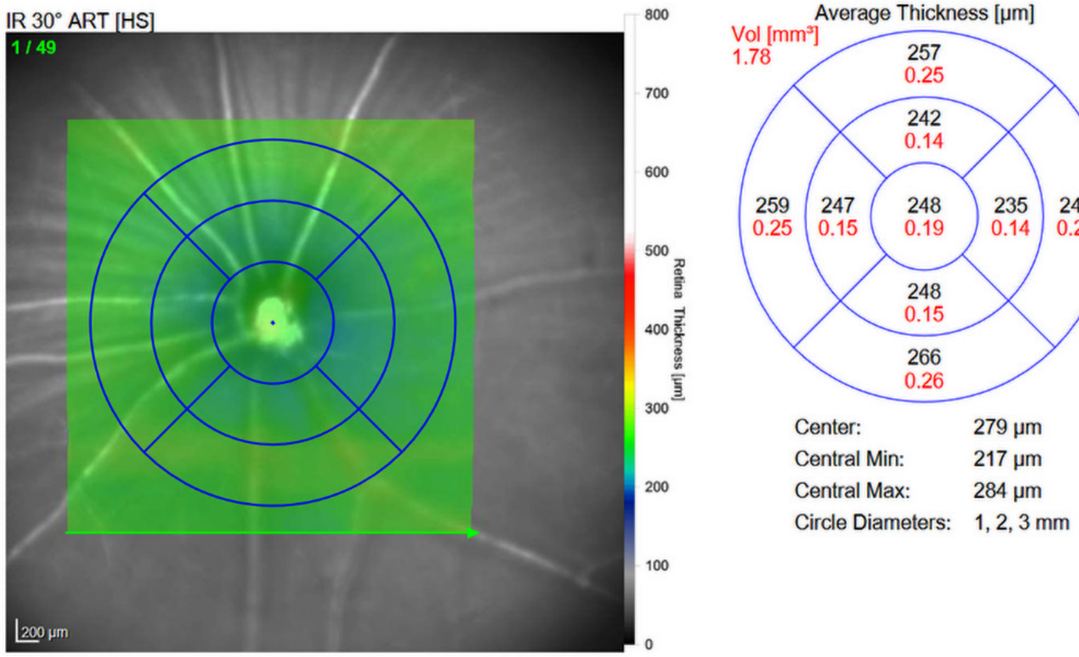

Figure S2: Example of macular thickness measurements obtained with SD-OCT system. Macular thickness map using circles of 1 mm, 2 mm, and 3 mm showing the mean thickness in each of the 9 subfields.
